# Association Between Autistic Traits in Preschool Children and Later Emotional/Behavioral Outcomes

**DOI:** 10.1007/s10803-017-3245-7

**Published:** 2017-08-07

**Authors:** Aya Saito, Andrew Stickley, Hideyuki Haraguchi, Hidetoshi Takahashi, Makoto Ishitobi, Yoko Kamio

**Affiliations:** 1 Department of Child and Adolescent Mental Health, National Institute of Mental Health, National Center of Neurology and Psychiatry (NCNP), 4-1-1 Ogawahigashi, Kodaira, Tokyo, 187-8553 Japan; 2 The Stockholm Center for Health and Social Change (SCOHOST), Södertörn University, 141 89 Huddinge, Sweden

**Keywords:** Autistic traits, Emotional/behavioral outcomes, Preschool children, Social Responsiveness Scale, Strengths and Difficulties Questionnaire

## Abstract

Although children with a greater number of autistic traits are likely to have other mental health problems, research on the association between earlier autistic traits in preschool children and later emotional/behavioral outcomes is scarce. Using data from 189 Japanese community-based children, this study examined whether autistic traits at age 5 were related to emotional/behavioral outcomes at age 7. The results showed that prior autistic traits were subsequently associated with all emotional/behavioral domains. After controlling for baseline emotional/behavioral scores autistic traits continued to predict later emotional symptoms and peer problems. This study highlights that in addition to clinical ASD, it is also important to focus on subthreshold autistic traits in preschool children for better subsequent emotional/behavioral outcomes.

## Introduction

Autism spectrum disorder (ASD) is a neurodevelopmental psychiatric disorder characterized by deficits in social communication and social interaction across multiple contexts, and the presence of restricted, repetitive patterns of behavior, interests, or activities (American Psychiatric Association 2013). Many previous studies have reported that additional mental health problems commonly occur in children with ASD (e.g., Leyfer et al. 2006; Lundström et al. 2015; Maskey et al. 2013; Simonoff et al. 2008, 2013; Totsika et al. 2011b). Simonoff et al. (2008) used data derived from an epidemiological sample to show that at age 10–14, 70% of children with ASD had at least one comorbid psychiatric disorder, and 41% had two or more disorders. Data from a population-based twin study similarly showed that a high percentage of children with ASD aged 9 and 12 years old also had other neuropsychiatric disorders, such as attention-deficit/hyperactivity disorder (ADHD) (51%), and learning disorders (LD) (35%) (Lichtenstein et al. 2010).

Among younger children with ASD, some studies have also found a high prevalence of other comorbid mental health problems. Salazar et al. (2015) reported that in a group of 101 children diagnosed with ASD at age 5–9 over 90% had at least one comorbid psychiatric disorder. Another recent study found that in a community sample of children with ASD aged 4–8 years old three-quarters had parent-reported emotional/behavioral problems, while two-thirds of these children who parents reported as being above the clinical cut-off for emotional/behavioral problems also had teacher-reported problems, as assessed by the Developmental Behavior Checklist (DBC; Einfeld and Tonge 2002) (Chandler et al. 2016). Preschool children with ASD as young as 2–4 years old have also been found to have increased levels of emotional/behavioral symptoms as assessed by the Child Behavior Checklist (CBCL; Achenbach and Rescorla 2000) (Georgiades et al. 2011). When using the Strengths and Difficulties Questionnaire (SDQ; Goodman 1997), ASD has been associated with increased odds for emotional/behavioral problems at age 5, even after controlling for intellectual disability and maternal mental health (Totsika et al. 2011a).

In DSM-5 (American Psychiatric Association 2013) a new diagnostic category name—ASD—was adopted where autistic traits are now more clearly identified as existing along a continuum. This followed in the wake of research which showed that quantitative autistic traits were continuously distributed even in the general population (reviewed in Constantino and Charman 2016). In connection with this, research has also highlighted that even when focusing on subthreshold ASD, children with a higher level of autistic traits have a greater risk of additional mental health problems (e.g., Lundström et al. 2011; Moriwaki and Kamio 2013). For example, in a population-based twin study, an association was reported between a greater number of autistic traits and having a higher risk of comorbid ADHD, anxiety, and conduct problems in children aged 9 and 12 years old (Lundström et al. 2011) as assessed by the Autism–Tics, ADHD, and other Co-morbidities inventory (A–TAC; Hansson et al. 2005; Larson et al. 2010). Research from Japan undertaken among regular classes at an elementary school and a junior high school has further shown that a higher number of autistic traits, assessed by the Social Responsiveness Scale (SRS; Constantino and Gruber 2005), is associated with SDQ assessed emotional and conduct problems (Moriwaki and Kamio 2013). The results from these studies thus suggest that as with ASD, there may also be a high rate of comorbid mental health problems among children with increased autistic traits. Furthermore, previous research that has focused on children and adolescents with anxiety disorders and/or mood disorders has also shown that they have higher levels of autistic traits (Pine et al. 2008; Towbin et al. 2005; van Steensel et al. 2013), which suggests that masked autistic traits might underlie more visible psychiatric problems. Given this, investigating mental health problems in children not only with clinical ASD but also with subthreshold ASD may be essential to gain a better understanding of how ASD symptoms are impacting on the emotional/behavioral outcomes of children.

The current study will examine how earlier autistic traits are associated with later emotional/behavioral outcomes in young children. Several previous studies have focused on the longitudinal relationship between earlier autistic traits and later mental health outcomes. For instance, using data from an approximately 1-year longitudinal follow-up study among ALSPAC cohort (Golding et al. 2001) children aged 7–8 years old, Skuse et al. (2009) showed that social communicative deficits, assessed by the parent-reported Social Communication Disorders Checklist (SCDC; Skuse et al. 2005), were significantly related to negative behavioral outcomes, assessed using all domains of the teacher-reported SDQ when simultaneously controlling for sex, IQ, and mother’s educational level. Hallett et al. (2010) conducted a study over a longer time span and found that autistic traits at age 7, assessed by the Childhood Autism Spectrum Test (CAST; Scott et al. 2002), contributed to the presence of internalizing problems at age 12, assessed by the emotional symptoms subscale of the SDQ. They also found that the magnitude of this association was bigger than that for the reverse relation between earlier internalizing problems and later autistic traits.

Compared to in school-age children, however, there has been almost no focus on the relationship between autistic traits in preschool children and later mental health outcomes apart from one study which used the Early Screening of Autistic Traits Questionnaire (ESAT; Dietz et al. 2006; Swinkels et al. 2006), to show that children with higher levels of autistic traits at age 14–15 months had more internalizing and externalizing problems at age 3 (as assessed by the CBCL) (Möricke et al. 2010). To the best of our knowledge, as yet, no studies have focused on the association between autistic traits in preschool children and emotional/behavioral outcomes in school-age children. Early detection and appropriate treatment of psychiatric problems in children are important since childhood psychiatric problems are associated with a higher risk of subsequent adverse outcomes even when the problems are subthreshold (Copeland et al. 2015).

Given the absence of research on the association between earlier autistic traits in preschool children and later emotional/behavioral outcomes in school-age children, the aim of this study was to investigate the association between earlier autistic traits at age 5 and later emotional/behavioral outcomes at age 7 in a cohort of community children. As SDQ scores have been shown to be moderately correlated in children between when they are in preschool and at age 7 (Lewis and Plomin 2015), in this study emotional/behavioral scores at age 5 were controlled for in order to disentangle the effects of autistic traits from those of baseline emotional/behavioral scores. By investigating the independent effects of autistic traits in addition to baseline emotional/behavioral scores for later emotional/behavioral outcomes, we will be able to assess the clinical significance of evaluating not only general mental health but also autistic traits in preschool children for identifying children at greater risk of later emotional/behavioral problems in school-age. In addition to examining the effects of total autistic traits, we will also focus on the separate effects of deficits in social communication and interaction (SCI) and restricted interests and repetitive behaviors (RRB) based on the DSM-5 classification (American Psychiatric Association 2013). Using the findings from previous studies among both younger (Möricke et al. 2010) and older children (Hallett et al. 2010; Skuse et al. 2009) as a starting point, we thus examined the hypothesis that a higher level of autistic traits at age 5 is correlated with higher levels of emotional symptoms, conduct problems, hyperactivity/inattention, peer problems, and lower levels of prosocial behavior at age 7, respectively.

## Methods

### Participants and Procedure

**Fig. 1:**
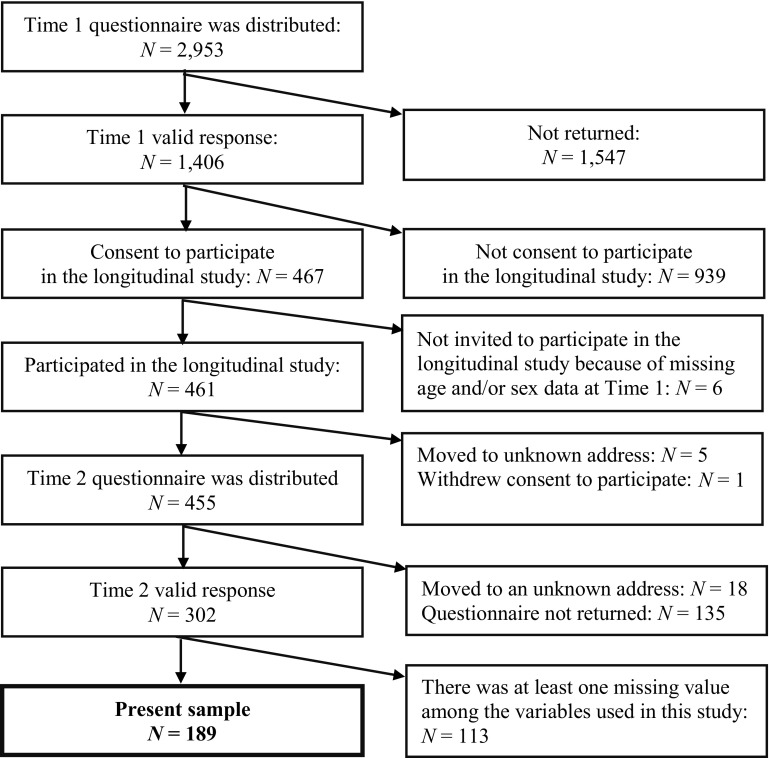
Flow diagram of the study participants

Data came from the Tama Children’s Survey (TCS), a cohort sample of community children from the Tama district of Tokyo, Japan. In the present study, data were analyzed from 189 children (107 boys) who had no missing information for autistic traits and mental health problems. A flow diagram of the participants is presented in Fig. 1.

In 2011, we invited 78 kindergartens and nursery schools in two cities in the Tama area of Tokyo, Japan, to participate in a survey, and obtained agreement from 64 institutions. In March 2012 (Time 1: age 5), a questionnaire and explanation sheet was distributed to 2953 families whose children were in classes for those aged 5 in these 64 institutions via their teachers. Each parent completed and returned the questionnaire voluntarily, which was considered as providing evidence of their informed consent to participate in this Time 1 questionnaire survey. There were 1406 valid responses, i.e. a response rate of 47.6%. The mean age of the children was 64.11 months (SD = 3.49 months). Families that participated in the Time 1 survey were asked whether they would consent to participate in a subsequent longitudinal study. Written informed consent to participate in the longitudinal study was obtained from 467 families. Six of the 467 families were excluded because of missing age and/or sex data at Time 1, resulting in 461 families participating in the longitudinal study. Comparing these 461 families against the Japanese general standard showed that the proportion of low-income families (under 2 million yen per year) among our participants was lower than the proportion in a survey conducted by the Ministry of Health, Labour and Welfare (2012), while the mean level of parental education measured in terms of mother’s and father’s years of schooling was higher than that reported by the United Nations Development Programme (2012). Moreover, using Time 1 survey data to compare the socioeconomic status (SES) of the 461 and 945 families who did and did not, respectively participate in the longitudinal study, showed that there was no difference in terms of household income [*t* (1366) = −0.826, *n.s*.] and level of education (years of schooling) among fathers [*t* (1321) = 0.819, *n.s*.], but that the level of education of mothers in the 461 families that agreed to participate in the cohort study was higher [*t* (1380) = 2.775, *p* < .01]. In September 2013, at Time 2 (age 7) questionnaires were distributed to 455 families, and returned by 302 families. One hundred and thirteen of the 302 children were excluded for missing one or more SRS and/or SDQ items, which meant that data from 189 children were analyzed in this study. At Time 1, the raters in all 189 families were mothers. At Time 2, the raters in 184 families were mothers, in 3 families fathers were raters, while in 2 families the sex of the rater was unknown.

### Instruments

#### Autistic Traits

Autistic traits at age 5 were measured with the parent-report Social Responsiveness Scale (SRS; Constantino and Gruber 2005). The SRS is a 65-item quantitative measure of autistic traits for children aged 4–18 years old. Each item is scored on a 4-point scale with total scores thus ranging from 0 to 195. The score distribution of the SRS is reported to be wide and continuous (Constantino and Todd 2003; Kamio et al. 2013a). In this study we used the Japanese version of the SRS, which like the original version (Constantino and Gruber 2012), has also been subsequently validated (Kamio et al. 2009, 2013a, 2013b; Moriwaki et al. 2011). The current version of the SRS (SRS-2) has been shown to have a two-factor structure (with one factor comprising 53 social communication interaction [SCI] items while the other comprises 12 restricted and repetitive behavior [RRB] items) that corresponds to DSM-5 criteria (Frazier et al. 2014a). Specifically, we used the 65-item total raw score, the 53-item SCI score, and the 12-item RRB score in the analyses (Cronbach’s α in this study = 0.93, 0.91, 0.82, respectively).

#### Emotional/Behavioral Outcomes

Emotional/behavioral scores at age 5 and age 7 were measured with the parent-report 25-item Strengths and Difficulties Questionnaire (SDQ; Goodman 1997). The SDQ is one of the most commonly used instruments for measuring emotional/behavioral outcomes in children. The validity and reliability of the scale have been confirmed in a previous study (Goodman 2001). It consists of five subscales measuring emotional symptoms, conduct problems, hyperactivity/inattention, peer problems, and prosocial behavior (Cronbach’s α in this study = 0.58–0.78). Each item is scored on a 3-point scale. The validity and reliability of the Japanese version of the SDQ has been demonstrated in previous studies (Matsuishi et al. 2008; Moriwaki and Kamio 2014).

#### Covariates

Children’s sex, household income, parental years of schooling and maternal depression have all been associated with mental health problems among children aged 5–7 years old in previous studies (Elberling et al. 2010; Lyons-Ruth et al. 1997). We measured these variables at Time 1, and included them in the analysis as control variables. Maternal depression was measured with a two question case-finding instrument (TQI). The TQI is a two question depression screening tool originally extracted from the Primary Care Evaluation of Mental Disorders Procedure (PRIME-MD) (Spitzer et al. 1994). The utility of the number of yes answers has been previously demonstrated for Japanese adults (Adachi et al. 2012).

### Ethical Considerations

This study was approved by the Ethics Committee of the National Center of Neurology and Psychiatry, Japan. After the Time 1 study, written informed consent to participate in the longitudinal study was obtained from the parents of each child participant.

### Data Analyses

In accordance with the results of a power analysis using G*Power 3 (Faul et al. 2007), to ensure that the participant numbers were sufficiently large, data were used from a combined sample of boys and girls in each analysis. First, we conducted independent *t* tests to compare SES, and the SRS and SDQ scores between the 189 families whose data were analyzed in the present study and the 272 families with full age and sex data that agreed to participate in the longitudinal study but whose data were not analyzed in the present study. For each *t* test if any of the 272 families were missing data for a particular outcome they were excluded from that specific analysis. Next, descriptive statistics of the study sample were calculated. Then, to investigate the association between autistic traits at age 5 and each emotional/behavioral outcome domain at age 5 and age 7, we calculated correlation coefficients between the SRS score at age 5 and each SDQ domain score at age 5 and age 7. Finally, hierarchical multiple regression analyses were conducted with emotional/behavioral outcomes at age 7 as the dependent variables. Children’s sex, household income, mother’s and father’s years of schooling, and maternal depression were entered as control variables in the first step (Step 1). Each emotional/behavioral score at age 5 was also entered as a control variable in the second step (Step 2). The total score, SCI score, and RRB score of the SRS at age 5 were entered in the third step, respectively (Step 3). A *p* value of <0.05 was considered as being statistically significant. All statistical analyses were performed using the computer software package IBM SPSS Statistics, version 22.

## Results

### Attrition

First, to determine whether there were any differences in terms of SES, and the SRS and SDQ scores at age 5 between the 189 families whose data were analyzed in the present study and the 272 families that agreed to participate in the longitudinal study but whose data were not analyzed in the present study, an analysis was conducted of the sample attrition. The results from an independent *t* test showed that there was no difference in household income [*t* (433) = −0.897, *n.s*.], father’s years of schooling [*t* (440) = −0.895, *n.s*.], and mother’s years of schooling [*t* (457) = −0.214, *n.s*.] between the two groups. There was also no difference in the SRS score [*t* (351) = −0.299, *n.s*.], or in the SDQ emotional symptoms [*t* (438) = −0.905, *n.s*.], conduct problems [*t* (438) = 1.240, *n.s*.], hyperactivity/inattention [*t* (438) = 1.020, *n.s*.], peer problems [*t* (438) = 0.477, *n.s*.], and prosocial behavior scores [*t* (438) = 1.423, *n.s*.]. The demographic characteristics of the participants and descriptive statistics of the dependent and independent variables used in this study are presented in Table 1.

**Table 1:** Sample characteristics and descriptive statistics of the independent and dependent variables (*N* = 189)

variables	Time 1		Time 2	
Children				
Age (mean ± SD years)	5.39 ± 0.49			
Sex				
Male	57.7%			
Female	42.3%			
Socioeconomic status (SES)				
Household income (million yen)				
Under 2	1.1%			
2 or more and less than 5	20.2%			
5 or more and less than 7	31.7%			
7 or more and less than 10	26.8%			
10 or more and less than 15	17.5%			
15 or more	2.7%			
Father’s years of schooling (mean ± SD years)	15.37 ± 2.14			
Mother’s years of schooling (mean ± SD years)	14.70 ± 1.65			
Maternal depression (number of “yes” answers)				
0	64.0%			
1	21.7%			
2	14.3%			

variables	Mean (SD)	Range	Mean (SD)	Range
Autistic traits (SRS)				
Total score	34.70 (19.31)	5–117		
Social communication/interaction (SCI)	29.69 (15.40)	4–92		
Restricted/repetitive behavior (RRB)	5.02 (4.61)	0–25		
Emotional/behavioral outcomes (SDQ)				
Emotional symptoms	1.66 (1.86)	0–10	1.67 (1.93)	0–9
Conduct problems	1.87 (1.57)	0–8	2.17 (1.68)	0–9
Hyperactivity/inattention	2.92 (2.25)	0–10	3.32 (2.09)	0–10
Peer problems	1.37 (1.62)	0–8	1.77 (1.82)	0–8
Prosocial behavior	6.62 (2.13)	0–10	6.11 (2.25)	0–10

1 million yen = ca. US $12,800 (in September 2012)

### Correlational Analyses

To investigate whether autistic traits at age 5 were correlated with each emotional/behavioral domain score at age 5 and age 7, correlation coefficients were computed. This showed that all autistic traits, i.e. the SCI, RRB, and total SRS scores at age 5 were significantly correlated with each SDQ domain score at age 5 with all the correlations being moderate to large (Table 2). In addition, the SCI, RRB, and total SRS scores at age 5 were also all significantly correlated with each SDQ domain score at age 7 although almost all of the correlation coefficient values were lower than those of the concurrent correlations (Table 2). There were large positive correlations between autistic traits at age 5 and peer problems at age 7. There were moderate positive correlations between autistic traits at age 5 and emotional symptoms and hyperactivity/inattention at age 7, and a moderate negative correlation between autistic traits at age 5 and prosocial behavior at age 7. Although earlier autistic traits were significantly correlated with later conduct problems, the association was weak.

**Table 2:** Correlations between autistic traits at age 5 and emotional/behavioral outcomes at age 5 and 7 (*N* = 189)

	Autistic traits at age 5		
	Total score	SCI	RRB
Emotional/behavioral outcomes at age 5			
Emotional symptoms	.50***	.49***	.45***
Conduct problems	.37***	.36***	.35***
Hyperactivity/inattention	.53***	.51***	.51***
Peer problems	.71***	.70***	.64***
Prosocial behavior	− .56***	− .58***	− .42***
Emotional/behavioral outcomes at age 7			
Emotional symptoms	.47***	.46***	.46***
Conduct problems	.22**	.20**	.25***
Hyperactivity/inattention	.40***	.37***	.43***
Peer problems	.58***	.57***	.54***
Prosocial behavior	− .46***	− .47***	− .33***

*SCI* social communication and interaction, *RRB* restricted interests and repetitive behaviors

**p < .01, ***p < .001

### Hierarchical Multiple Regression Analyses

To investigate whether autistic traits at age 5 were possible predictors of each emotional/behavioral outcome domain at age 7 after controlling for each domain score at age 5, hierarchical multiple regression analyses were conducted. The presence of multicollinearity was checked using the variance inflation factor (VIF) statistic. A VIF value greater than 10 is a common threshold for indicating severe multicollinearity (Chatterjee and Price 1990; O’Brien 2007). As the VIF was 2.18 or under for all of our predictors this indicated that multicollinearity was not a problem in the current study.

In the models predicting emotional symptoms and peer problems, in Step 2 when these specific age 5 domain scores were added to the analysis they were strongly associated with the same domain scores at age 7. In addition, the coefficients of determination also increased significantly when autistic traits were included in the analysis in Step 3 (Tables 3, 4, 5). Although an association between the same emotional/behavioral domain scores at different ages is expected, autistic traits at age 5 were found to be independent and significant predictors of emotional/behavioral outcomes at age 7. This predictive ability was commonly observed for the SRS total score, and the separate SCI and RRB scores, respectively. In the analyses predicting conduct problems, hyperactivity/inattention, and prosocial behavior, the inclusion of autistic traits in Step 3 did not result in a significant increase in the coefficient of determination in any of the models That is, autistic traits at age 5 (i.e. the autistic traits total score, and the SCI and RRB scores) were not significant predictors of these particular outcomes at age 7 after controlling for these variables’ domain scores at age 5 (Tables 3, 4, 5).

**Table 3:** Hierarchical multiple regression analysis of the contribution of sex, SES, maternal depression, emotional/behavioral baseline scores, and the autistic traits total score at age 5 to emotional/behavioral outcomes at age 7 (*N* = 189)

	Emotional symptoms				Conduct problems				Hyperactivity/inattention				Peer problems				Prosocial behavior			
	B	SE B	β	Δ*R* ^2^	B	SE B	β	Δ*R* ^2^	B	SE B	β	Δ*R* ^2^	B	SE B	β	Δ*R* ^2^	B	SE B	β	Δ*R* ^2^
Step1				.07*				.09**				.12**				.06				.06*
Sex	.07	.23	.02		.13	.20	.04		−.08	.23	−.02		−.01	.21	−.00		.22	.26	.05	
Household income	−.06	.09	−.04		−.04	.08	−.03		−.05	.09	−.03		−.07	.08	−.05		−.09	.10	−.05	
Father’s education (years)	−.06	.06	−.06		.01	.06	.01		−.01	.06	−.01		.06	.06	.07		−.05	.07	−.05	
Mother’s education (years)	−.03	.08	−.03		−.05	.07	−.04		−.10	.08	−.08		−.13	.07	−.12		.04	.09	.03	
Maternal depression	.09	.16	.03		.15	.15	.07		.31	.16	.11		.15	.19	.06		−.33	.18	−.11	
Step2				.31***				.28***				.35***				.33***				.34***
Each emotional/behavioral domain score at age 5	.49	.07	.47***		.62	.07	.58***		.57	.06	.61***		.44	.09	.39***		.57	.08	.54***	
Step3				.04**				.00				.00				.04**				.01
Autistic traits total score at age 5	.02	.01	.23**		−.00	.01	−.01		.00	.01	.04		.03	.01	.29**		−.01	.01	−.12	
*R* ^2^	.42				.37				.47				.43				.41			
*adj R* ^2^	.39				.34				.45				.40				.39			

B, SE B, β, *R* ^2^, and *adj R* ^2^ are for Step 3 when all predictors are included in the analysis

*p < .05, **p < .01, ***p < .001

**Table 4:** Hierarchical multiple regression analysis of the contribution of sex, SES, maternal depression, emotional/behavioral baseline scores, and the autistic traits SCI score at age 5 to emotional/behavioral outcomes at age 7 (*N* = 189)

	Emotional symptoms				Conduct problems				Hyperactivity/inattention				Peer problems				Prosocial behavior			
	B	SE B	β	Δ*R* ^2^	B	SE B	β	Δ*R* ^2^	B	SE B	β	Δ*R* ^2^	B	SE B	β	Δ*R* ^2^	B	SE B	β	Δ*R* ^2^
Step1				.07*				.09**				.12**				.06				.06*
Sex	.05	.23	.01		.13	.20	.04		−.09	.23	−.02		.05	.23	.01		.23	.26	.05	
Household income	−.06	.09	−.04		−.04	.08	−.03		−.05	.09	−.03		−.06	.09	−.04		−.08	.10	−.05	
Father’s education (years)	−.05	.06	−.06		.01	.06	.01		−.01	.06	−.01		−.05	.06	−.06		−.05	.07	−.05	
Mother’s education (years)	−.03	.08	−.03		−.05	.07	−.05		−.10	.08	−.08		−.03	.08	−.03		.04	.09	.03	
Maternal depression	.11	.16	.04		.15	.15	.07		.32	.16	.11		.11	.16	.04		−.33	.18	−.11	
Step2				.31***				.28***				.35***				.33***				.34***
Each emotional/behavioral domain score at age 5	.50	.07	.48***		.62	.07	.58***		.57	.06	.62***		.50	.07	.48***		.56	.08	.53***	
Step3				.03**				.00				.00				.03**				.01
Autistic traits SCI score at age 5	.03	.01	.21**		−.00	.01	−.02		.00	.01	.02		.03	.01	.21**		−.02	.01	−.14	
*R* ^2^	.41				.37				.47				.41				.42			
*adj R* ^2^	.39				.34				.45				.39				.40			

B, SE B, β, *R* ^2^, and *adj R* ^2^are for Step 3 when all predictors are included in the analysis

*SCI* social communication and interaction

*p < .05, **p < .01, ***p < .001

**Table 5:** Hierarchical multiple regression analysis of the contribution of sex, SES, maternal depression, emotional/behavioral baseline scores, and the autistic traits RRB score at age 5 to emotional/behavioral outcomes at age 7 (*N* = 189)

	Emotional symptoms				Conduct problems				Hyperactivity/inattention				Peer problems				Prosocial behavior			
	B	SE B	β	Δ*R* ^2^	B	SE B	β	Δ*R* ^2^	B	SE B	β	Δ*R* ^2^	B	SE B	β	Δ*R* ^2^	B	SE B	β	Δ*R* ^2^
Step1				.07*				.09**				.12**				.06				.06*
Sex	.10	.23	.03		.15	.21	.04		−.06	.23	−.01		.10	.23	.03		.22	.27	.05	
Household income	−.04	.09	−.03		−.04	.08	−.03		.06	.09	−.04		−.04	.09	−.03		−.11	.10	−.06	
Father’s education (years)	−.06	.06	−.07		.01	.06	.01		−.01	.06	−.01		−.06	.06	−.07		−.06	.07	−.05	
Mother’s education (years)	−.02	.08	−.02		−.04	.07	−.04		−.09	.08	−.07		−.02	.08	−.02		.05	.09	.04	
Maternal depression	.06	.16	.02		.13	.15	.06		.28	.16	.10		.06	.16	.02		−.37	.19	−.12	
Step2				.31***				.28***				.35***				.33***				.34***
Each emotional/behavioral domain score at age 5	.50	.07	.48***		.60	.07	.56***		.54	.06	.58***		.50	.07	.48***		.62	.07	.59***	
Step3				.04**				.00				.01				.04**				.00
Autistic traits RRB score at age5	.10	.03	.23**		.02	.02	.04		.05	.03	.10		.10	.03	.23**		−.02	.03	−.04	
*R* ^2^	.42				.37				.48				.42				.41			
*adj R* ^2^	.40				.34				.46				.40				.38			

B, SE B, β, *R* ^2^, and *adj R* ^2^are for Step 3 when all predictors are included in the analysis

*RRB* restricted interests and repetitive behaviors

*p < .05, **p < .01, ***p < .001

## Discussion

The purpose of this study was to examine the association between autistic traits at age 5 and various emotional/behavioral outcome domains at age 7 in a cohort of community children. Results indicated that prior autistic traits at age 5 were subsequently associated with all emotional/behavioral outcome domains at age 7. Furthermore, earlier autistic traits continued to predict later emotional symptoms and peer problems even after controlling for baseline emotional/behavioral scores.

In the correlation analysis, preschoolers with a greater number of autistic traits had a higher risk of experiencing various emotional/behavioral problems including emotional symptoms, conduct problems, hyperactivity/inattention, peer problems, and of exhibiting less prosocial behavior. These results are consistent with our hypothesis and the results from previous studies which have shown an association between earlier autistic traits and later emotional/behavioral problems in other developmental stages, i.e. in children aged 7–12 years old (Hallett et al. 2010), and from 14 to 15 months to 3 years old (Möricke et al. 2010). This study builds on the findings of these earlier studies by showing that these associations are also found between preschool children at age 5 and school-age children at age 7. The present study also extends an earlier research finding of an association between social communicative deficits and behavior problems in children aged 7–8 (Skuse et al. 2009), by demonstrating that in addition to social communication and interaction difficulties, restricted interests and repetitive behaviors are also related to later emotional/behavioral outcomes.

Concerning the possible association between earlier autistic traits at age 5 and later emotional/behavioral outcomes at age 7, after controlling for the emotional/behavioral baseline score, results varied across the different emotional/behavioral outcome domains. For all domains, emotional/behavioral scores at age 5 were the strongest predictors of emotional/behavioral outcomes at age 7. This suggests that children who already have emotional/behavioral problems at age 5 have a much greater risk of later emotional/behavioral problems, so it is important to identify these children and to follow them up appropriately. For conduct problems, hyperactivity/inattention, and prosocial behavior, there was no association between autistic traits at age 5 and these outcomes at age 7 after controlling for the baseline score of each domain. This indicates that the predictive power of each of these domains at age 5 was large and that the level of autistic traits at age 5 was not important for these particular emotional/behavioral outcomes. In contrast, for emotional symptoms and peer problems, autistic traits at age 5 were a small but significant predictor of these outcomes at age 7 even after considering the effects of these domain scores at age 5.

The most notable finding of this study was that autistic traits at age 5 predicted emotional symptoms at age 7 even after controlling for baseline emotional symptoms at age 5. Interestingly, this result accords with the finding of Hallett et al. (2010) who showed that autistic traits at age 7 predicted later emotional symptoms at age 12. In twin studies it has previously been suggested that there are modest levels of phenotypic overlap between children’s autistic traits and internalizing traits which might be explained in some part by shared genetic factors (Hallett et al. 2009), while another study showed that common genetic effects accounted for most of the correlation between autistic traits and anxiety traits (Lundström et al. 2011). Despite this, we cannot conclude that there is a causal relationship between autistic traits at age 5 and emotional symptoms at age 7 as it is possible that emotional symptoms and autistic traits might both be caused by some, as yet, unidentified common underlying factor. Alternatively, it is possible that autistic traits may modify the developmental course of emotional symptoms from age 5 to age 7. Specifically, children with higher levels of autistic traits might not be able to seek social support properly in a fear or anxiety evoking situation because of deficits in social communication and interaction, or their emotional symptoms may not be noticed by others because of atypical emotional expression, resulting in a failure to obtain social support. It is also possible that children with higher levels of autistic traits may be unable to avoid anxiety-evoking situations because of the persistence of existing routines, resulting in increasing anxiety. In addition, entrance into elementary school may be stressful for young children because greater behavioral demands are placed on them, while cross-cultural differences in school culture might have a negative effect. In particular, as the average class size in Japanese primary school is larger than in many other countries (Organisation for Economic Co-operation and Development 2016), the environmental change that occurs at this age when moving from kindergarten to elementary school might have constituted a significantly greater burden for the children in this study. The association observed might therefore be explained by the fact that children with a greater number of autistic traits might be especially vulnerable to such an environmental change, which may result in them having elevated levels of anxiety.

Regarding peer problems, the autistic traits total score, and the SCI and RRB scores were all predictive of later peer problems in this study. This finding extends the result of an earlier cross-sectional study which showed an association between autistic traits and negative peer relationships including an inability to make friends and maintain relationships, and problems with peers including shyness, bullying, and victimization in children in grades 1–8 in Taiwan (Hsiao et al. 2013). Several previous studies have also reported that ASD is linked to a greater risk of experiencing school-based peer victimization (reviewed in Maïano et al. 2016) and a greater degree of loneliness (Lasgaard et al. 2010), and that victimization or loneliness might be risk factors for peer problems.

### Study Strengths and Limitations

To the best of our knowledge, this is the first study to examine the relation between earlier autistic traits in preschool children and later emotional/behavioral outcomes in school-age children. The use of internationally comparable measures—the SRS and SDQ—which have been validated in many parts of the world, including Japan, was also a strength of the present study.

It should be noted however, that a few of the SRS items have a similar description to some of the SDQ peer problems and prosocial behavior subscale items even though the SRS and SDQ were developed to measure different constructs. As a result of this children who have high SRS scores on some items may also have higher scores on some related SDQ items. Having said this, some overlap between what is measured by the SRS and what is measured by a general psychopathology scale may be unavoidable as several cross-sectional studies have previously highlighted. For example, Hus et al. (2013) reported a small to moderate association between internalizing and externalizing behavior problem scores assessed by the CBCL and the SRS score among both ASD probands and non-ASD siblings. In addition, Frazier et al. (2014b) indicated that ADHD, anxiety disorder, or intellectual disability were all small but significant predictors of the SRS score even after considering an ASD diagnosis. In our study, a concurrent association between the SRS score and all of the SDQ domain scores was also found, however, our main interest in this study was the longitudinal association rather than the concurrent association. In our multiple regression analyses, we included not only the SRS score at age 5 but also each SDQ domain baseline score at age 5 as independent variables in order to disentangle the effects of autistic traits from the effects of emotional/behavioral problems for later emotional/behavioral outcomes.

This study has several other limitations. Following the lead of a previous study (Möricke et al. 2010), and due to our small sample size and desire to ensure sufficient statistical power, in this study we analyzed a combined sample of boys and girls. Little is known about sex differences in the clinical characteristics of children with ASD or higher autistic traits. Previous studies have reported conflicting findings. For instance, regarding the association between ASD and comorbid emotional problems such as anxiety, worry or depression, although several studies reported that there were no sex differences in these associations in children and adolescents (Holtmann et al. 2007; Worley and Matson 2011), another study found that among toddlers, girls with ASD symptoms exhibited more emotional problems than boys (Hartley and Sikora 2009). Given this, it is important that future studies should use a larger sample size so that possible sex differences in these associations in this age band can be examined. It is also possible that the results of this study might not be representative for the whole of Japan as survey data were obtained from only one region in Tokyo, and some of the sample characteristics differ from the mean values observed in representative samples of the Japanese population. The high rate of attrition is also a limitation of this study. In addition, since all measurement data were obtained from only the parents of each child and not from other adults such as teachers, we could not determine whether there was a rater effect in the observed associations. Finally, there was no information about life events such as the children’s experience in school or at home. In future, to facilitate more comprehensive research, data should be obtained from not only parents but also from teachers and include more detailed information on environmental factors.

### Clinical Implications

The finding that earlier autistic traits are a risk factor for later emotional and peer problems may have clinical implications. First, in addition to general mental health problems, the results of this study suggest that it might also be beneficial to evaluate autistic traits in preschool children in order to identify those children who have a greater risk of experiencing later mental health problems, particularly emotional problems, as paying careful attention to individuals with higher levels of autistic traits and emotional symptoms will help determine the need for early intervention and the prevention of later emotional problems. If emotional problems in children are not treated appropriately, it can result in extremely detrimental outcomes such as poorer academic functioning (Edelsohn et al. 1992), school adjustment (Yoleri 2013), and worse adaptive functioning (Ialongo et al. 1995). In addition, increased emotional symptoms in children have also been linked to more serious later disorders such as major depression (Pine et al. 1999) and mood disorders (Roza et al. 2003). Similarly, as peer problems in childhood have been associated with serious adjustment problems in later life (reviewed in Parker and Asher 1987), then paying careful attention to preschool children with higher levels of autistic traits and peer problems may also be important for identifying deficits in children’s social skills or to ensure they receive appropriate treatment.

Second, clinicians should check autistic symptoms in children with emotional problems even if the chief complaint is not regarded as being associated with autism because there may be underlying autistic traits at a subclinical/clinical level which also need to be addressed, especially as several earlier studies reported that children and adolescents with anxiety and/or mood disorders also had higher levels of autistic traits even without having an ASD diagnosis (Pine et al. 2008; Towbin et al. 2005; van Steensel et al. 2013). In addition, teachers and family members need to be made aware of the possibility that higher levels of autistic traits can sometimes underlie children’s peer problems even if they are not diagnosed with ASD, as it may encourage help-seeking behavior.

In conclusion, this study demonstrated an association between age 5 autistic traits and age 7 emotional/behavioral outcomes. More specifically, autistic traits at age 5 predicted emotional symptoms and peer problems at age 7 after controlling for each of these baseline scores at age 5. This highlights the importance of not only focusing on clinical cases of ASD among preschool children but also of examining subthreshold autistic traits in this age group in order to ensure better subsequent emotional/behavioral outcomes.
